# Association of fibrinogen to albumin ratio with sepsis-associated acute kidney injury: A retrospective cohort study based on the MIMIC-IV database

**DOI:** 10.1371/journal.pone.0343549

**Published:** 2026-03-06

**Authors:** Tuan Li, Muyan Diao, Feng Lu, Zhuojian Zeng, Zhiwen Chen, Shengyuan Su, Yuehui Zhang

**Affiliations:** 1 Department of Critical Care Medicine, The Second Affiliated Hospital of Shenzhen University, Shenzhen, Guangdong, China; 2 Shenzhen International Travel Healthcare Center (Shenzhen Customs District Port Outpatient Clinics), Shenzhen, Guangdong, China; 3 Department of Critical Care Medicine, Shenzhen Bao'an Clinical Medical College of Guangdong Medical University, Shenzhen, Guangdong, China; Azienda Ospedaliero Universitaria Careggi, ITALY

## Abstract

**Purpose:**

Sepsis-associated acute kidney injury (SA-AKI) is a critical complication associated with negative outcomes. However, the effective prevention of SA-AKI is limited. This retrospective cohort study, which used the MIMIC-IV database, investigated the association between fibrinogen-to-albumin ratio (FAR) and SA-AKI.

**Materials and methods:**

The retrospective cohort study involved 1,771 sepsis patients in MIMIC-IV database. Multivariable logistic and Cox regression models were used to estimate ORs/HRs with 95% CIs for incident SA-AKI. Sensitivity analyses, including stratified analyses and RCS curve, assessed the strength of the association. The predictive performance was compared to other marker using ROC curves and AUCs.

**Results:**

An elevated FAR level (≥110.74) was found to be associated with an elevated risk of SA-AKI (adjusted OR 1.55, 95%CI 1.11–2.18, P = 0.011), but the association was timing, it reached statistical significance only when SA-AKI occurred after ICU day 3 (adjusted HR 5.17, 95%CI 1.81–14.72, P = 0.002). Subgroup analyses indicated that chronic obstructive pulmonary disease (COPD) and hypertension interacted in this association. In sepsis patients without COPD and hypertension, high FAR (≥110.74) was linked to SA-AKI (adjusted OR 2.31, 95%CI 1.40–3.82, P = 0.001), with statistical significance also occurring 3 days after ICU admission (adjusted HR 8.57, 95%CI 1.88–38.94, P = 0.005). The RCS curve showed a linear relationship between FAR and SA-AKI (P for non-linearity: 0.415). ROC analyses showed that FAR combined with SOFA slightly outperformed SOFA alone (AUC 0.697 vs. 0.678, P = 0.004).

**Conclusions:**

An elevated FAR level was associated with an increased incidence of SA-AKI in patients without COPD and hypertension. However, this association reached statistical significance only when SA-AKI occurred after ICU day 3. Further research is needed to investigate this association.

## Introduction

Sepsis-associated acute kidney injury (SA-AKI) is defined as an acute deterioration of renal function occurring in the context of sepsis. It is an early, common, life-threatening complication and is defined by both Sepsis-3 and Kidney Disease: Improving Global Outcomes (KDIGO) criteria [1,2]. It is linked to high mortality, more cardiovascular events, and substantial costs. However, its exact mechanisms have not been fully elucidated [2,3]. The ability to early detect SA-AKI is currently inadequate. Hence, exploring and identifying relevant clinical indicators is vital for improving this prognosis.

FAR (fibrinogen-to-albumin ratio), currently recognized as a novel biomarker, has been confirmed to be associated with an increased risk of cancer mortality, peritonitis-related sepsis mortality, mortality in peritoneal dialysis patients, and acute coronary syndrome et al. [4–7]. However, evidence regarding the association between FAR and SA‑AKI remains limited. Therefore, this study aimed to evaluate the relationship between FAR and SA‑AKI.

## Materials and methods

This retrospective cohort study adhered to the strengthening the reporting of observational studies in epidemiology (STROBE) and reporting of studies conducted using observational routinely-collected data (RECORD) guidelines [8,9], and used de-identified data from the Medical Information Mart for Intensive Care (MIMIC)-IV database version 2.0 (2008–2019, via Physionet) [10,11]. The MIMIC-IV database received approval from the Massachusetts Institute of Technology (Cambridge, MA) and Beth Israel Deaconess Medical Center (Boston, MA), with informed consent originally obtained for data collection. Therefore, the ethical approval and informed consent were waived for this manuscript. Data use permissions were obtained (certification number: 46498677, 44131633, 54492745).

### Study patients and data extraction

**Fig 1:**
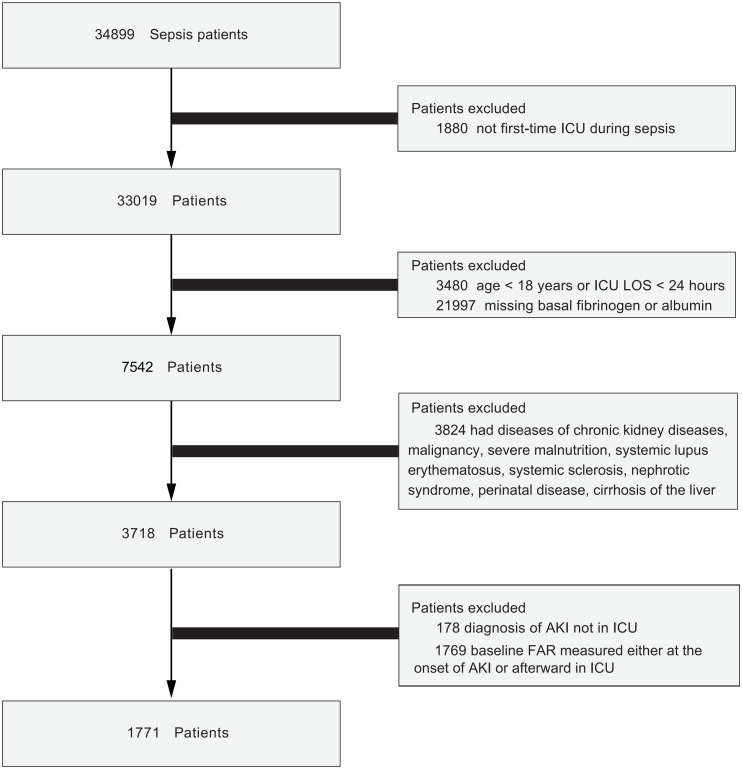
Flowchart of participants through the study.

Patients meeting the Sepsis-3 criteria [12] were included. Diagnosis of SA-AKI required meeting both Sepsis-3 and KDIGO criteria [3,13]. Exclusion criteria included: (1) only the first intensive care unit (ICU) admission per hospitalization included; subsequent stays excluded (n = 1880) (2) age < 18 years or ICU stay <24 hours excluded (n = 3480), (3) missing the first-time FAR value in ICU stay (n = 21997) (S1 Table), (4) patients with diseases such as chronic kidney diseases (CKD), malignancy, severe malnutrition, systemic lupus erythematosus, systemic sclerosis, nephrotic syndrome, perinatal disease and severe liver dysfunction excluded (n = 3824), as these diseases may affect FAR level. “Severe liver dysfunction” was referred to conditions such as alcoholic cirrhosis of liver, cirrhosis of liver without mention of alcohol, biliary cirrhosis, alcoholic fibrosis and sclerosis of liver, alcoholic cirrhosis of liver without ascites, alcoholic cirrhosis of liver with ascites, hepatic sclerosis and primary biliary cirrhosis, (5) SA-AKI diagnosed outside the ICU excluded (n = 178), (6) the first-time FAR value measured either at the onset of SA-AKI or afterward excluded (n = 1769) (Fig 1).

Our primary independent variable was the first-time FAR (the ratio of fibrinogen to albumin) in ICU stay.The endpoint was the occurrence of SA-AKI in ICU. The data were extracted from MIMIC-IV (version 2.0) using PostgreSQL 11.2. Patients’ demographics and comorbidities, including age, gender, race, chronic obstructive pulmonary disease (COPD), hypertension, diabetes, heart failure were collected. Other first laboratory parameters on ICU admission were also selected for analysis, including creatinine, platelets, white blood cell count (WBC), prothrombin time (PT), activated partial thromboplastin time (APTT), glucose. Furthermore, we collected mean blood pressure (MBP), charlson comorbidity index, sepsis-related organ failure assessment (SOFA) score, simplified acute physiology score II (SAPSII score). The intervention procedures included vasopressor use (any of dobutamine, dopamine, epinephrine, norepinephrine, milrinone, phenylephrine) and mechanical ventilation (MV). Relevant outcomes included renal replacement therapy (RRT); hospital and ICU length of stay; in-hospital/ICU mortality, in 30-day mortality; disseminated intravascular coagulation (DIC) and acute kidney injury (AKI) stage.

### Statistical analysis

Skewed variables were log-transformed to minimize outlier effects. Missing data were addressed using multiple imputation by chained equations (mice in R; three imputations) (S2 Table). Patients were stratified into FAR tertiles: low-FAR group (FAR < 64.82), medium-FAR group (64.82 ≤ FAR < 110.74) and high-FAR group (FAR ≥ 110.74). Continuous data were presented as mean ± standard deviation (SD) or median (interquartile range, IQR); categorical variables as number (%). Differences across tertiles were examined with the Kruskal–Wallis or one-way ANOVA test (continuous variables) and the Chi-square test (categorical variables). The multi-collinearity was assessed using variance inflation factors (VIF>=5) and Pearson/Spearman correlations (two-tailed).

Associations were evaluated by logistic and Cox regression models, with the low-FAR group as reference. Logistic regression models were built sequentially: an unadjusted crude model; Model I adjusted for age, gender, and race; Model II additionally adjusted for comorbidities and Model III further adjusted for vasopressor use, MV, DIC, SOFA score, SAPSII score, creatinine, PT, APTT, platelets, WBC and MBP. Temporal relationships between FAR and SA‑AKI were examined in Cox regression models.

Sensitivity analyses were conducted to evaluate the robustness of this association. These included stratified analyses by age, DIC, comorbidities and vasopressor use, with interaction testing; significant interactions were further explored by using logistic and Cox regression models. A restricted cubic spline (RCS) was applied to examine the continuous relationship between FAR and SA-AKI. Predictive performance was assessed using receiver operator characteristic curves (ROCs) and the corresponding areas under curve (AUCs) for FAR and other markers.

All statistical analyses were performed using IBM SPSS Statistics version 26.0, and the Free Statistics software versions 1.7 (http://www.clinicalscientists.cn/freestatistics). Significance was set at P < 0.05.

## Results

### Study cohort and patient characteristics

**Fig 2:**
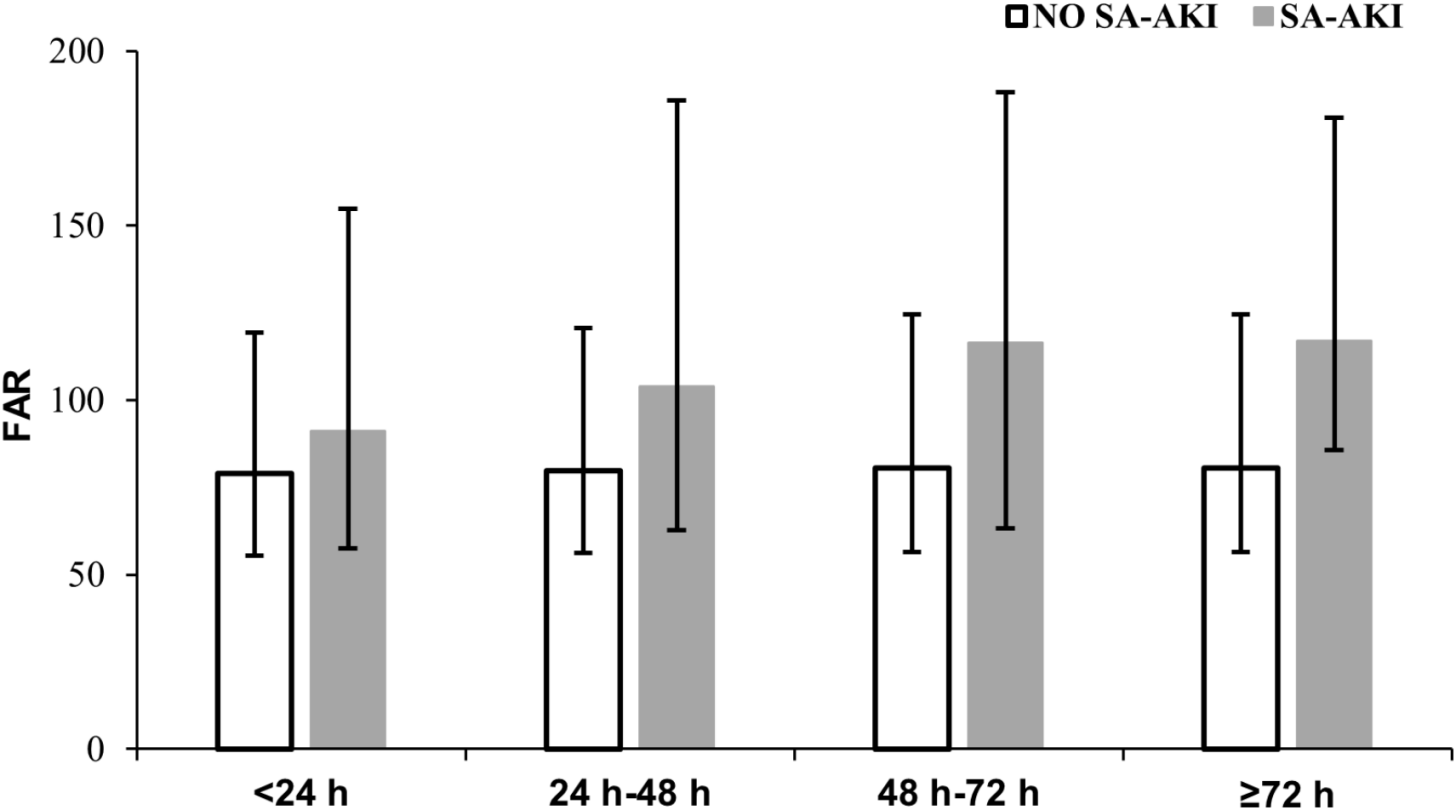
Bar chart of FAR values based on time windows.

The study flow chart was presented in Fig 1. This cohort included 1,771 patients with sepsis. Patients’ detailed characteristics according to FAR tertiles were shown in Table 1. Among those participants, 722 were women and 1,049 were men, with a median age of 61.0 (IQR 46.0–73.0) years. During their ICU stay, 568 patients (32.1%) developed SA-AKI. The incidence of SA-AKI was highest in the high-FAR group (242 patients, 40.9%), compared with the medium-FAR (157 patients, 26.6%) and low-FAR groups (169 patients, 28.6%) (P < 0.001), suggesting a potential association between higher FAR and SA-AKI. In addition, patients in the high-FAR group were older and were more likely to suffer from diabetes, heart failure and had longer ICU and hospital stays as well as higher SOFA and SAPSII score. Across different time windows, FAR values were consistently higher in patients with SA-AKI than in those without (Table 2, Fig 2).

**Table 1:** Baseline characteristics of participants.

Variables	Total	FAR < 64.82	64.82 ≤ FAR < 110.74	FAR ≥ 110.74	P-value
	(n = 1771)	(n = 590)	(n = 590)	(n = 591)	
Age (yr), median (IQR)	61.0 (46.0, 73.0)	57.1 (39.9, 69.6)	62.3 (47.7, 74.8)	62.3 (49.8, 75.3)	<0.001
Race, n (%)					0.008
White	1079 (60.9)	349 (59.2)	363 (61.5)	367 (62.1)	
Black	122 (6.9)	37 (6.3)	29 (4.9)	56 (9.5)	
Other	570 (32.2)	204 (34.6)	198 (33.6)	168 (28.4)	
Gender, n (%)					<0.001
Female	722 (40.8)	206 (34.9)	246 (41.7)	270 (45.7)	
Male	1049 (59.2)	384 (65.1)	344 (58.3)	321 (54.3)	
Comorbidities, n (%)					
COPD	104 (5.9)	26 (4.4)	36 (6.1)	42 (7.1)	0.137
Hypertension	821 (46.4)	268 (45.4)	282 (47.8)	271 (45.9)	0.684
Diabetes	376 (21.2)	86 (14.6)	144 (24.4)	146 (24.7)	<0.001
Heart failure	373 (21.1)	88 (14.9)	138 (23.4)	147 (24.9)	<0.001
Laboratory parameters					
Far, median (IQR)	84.5 (56.4, 134.4)	47.9 (38.8, 56.4)	84.5 (73.8, 96.2)	173.8 (134.4, 233.1)	<0.001
Fibrinogen (mg/dL), median (IQR)	272.0 (190.0, 411.0)	164.0 (130.0, 203.8)	277.0 (238.0, 323.0)	491.0 (397.0, 643.0)	<0.001
Albumin (g/dL), mean (SD)	3.2 ± 0.7	3.5 ± 0.7	3.3 ± 0.7	2.8 ± 0.6	<0.001
Creatinine (mg/dL), median (IQR)	1.0 (0.8, 1.3)	1.0 (0.8, 1.2)	1.0 (0.8, 1.3)	1.0 (0.7, 1.5)	0.364
Platelets (K/uL), median (IQR)	196.0 (134.0, 269.0)	192.5 (137.0, 253.8)	198.0 (141.0, 272.8)	199.0 (125.0, 286.0)	0.133
WBC (K/uL), median (IQR)	11.8 (8.1, 17.1)	11.5 (8.1, 16.5)	11.6 (8.0, 16.4)	12.4 (8.2, 18.7)	0.149
PT, median (IQR)	14.0 (12.3, 17.7)	13.6 (11.9, 18.1)	13.3 (12.0, 16.6)	14.9 (13.0, 18.4)	<0.001
APTT, median (IQR)	30.8 (26.6, 39.4)	32.2 (27.3, 45.0)	29.5 (25.7, 35.5)	31.1 (26.6, 37.8)	<0.001
Glucose (mg/dL), median (IQR)	135.0 (108.0, 178.0)	133.0 (108.0, 172.8)	139.0 (111.0, 184.8)	133.0 (107.0, 174.5)	0.055
MBP (mmHg), mean (SD)	84.4 ± 19.5	85.5 ± 19.5	85.8 ± 19.3	81.9 ± 19.4	<0.001
Charlson score, median (IQR)	4.0 (2.0, 6.0)	4.0 (2.0, 5.0)	4.0 (3.0, 6.0)	4.0 (2.0, 6.0)	< 0.001
SOFA score, median (IQR)	3.0 (2.0, 5.0)	3.0 (2.0, 5.0)	3.0 (2.0, 4.0)	4.0 (2.0, 5.0)	0.005
SAPSII score, median (IQR)	37.0 (29.0, 47.0)	36.0 (28.2, 46.0)	36.0 (29.0, 46.0)	39.0 (30.0, 50.0)	<0.001
Vasopressor use, n (%)	1090 (61.5)	399 (67.6)	325 (55.1)	366 (61.9)	<0.001
MV use, n (%)	1397 (78.9)	516 (87.5)	446 (75.6)	435 (73.6)	<0.001
Clinical outcomes					
RRT, n (%)	149 (8.4)	63 (10.7)	33 (5.6)	53 (9.0)	0.006
Hospital LOS (days), median (IQR)	9.7 (5.8, 17.9)	8.9 (5.2, 17.0)	9.5 (5.8, 16.7)	10.8 (6.6, 20.0)	0.001
ICU LOS (days), median (IQR)	4.7 (2.5, 9.8)	4.4 (2.4, 9.5)	4.6 (2.4, 9.4)	5.0 (2.7, 10.1)	0.041
ICU Mortality, n (%)	289 (16.3)	106 (18.0)	87 (14.7)	96 (16.2)	0.326
Hospital Mortality, n (%)	314 (17.7)	110 (18.6)	96 (16.3)	108 (18.3)	0.517
30-day Mortality, n (%)	385 (21.7)	126 (21.4)	124 (21.0)	135 (22.8)	0.721
DIC, n (%)	60 (3.4)	33 (5.6)	14 (2.4)	13 (2.2)	0.001
AKI, n (%)	568 (32.1)	169 (28.6)	157 (26.6)	242 (40.9)	<0.001
AKI stage, n (%)					0.335
1	429 (75.5)	131 (77.5)	120 (76.4)	178 (73.6)	
2	130 (22.9)	35 (20.7)	37 (23.6)	58 (24.0)	
3	9 (1.6)	3 (1.8)	0 (0.0)	6 (2.5)	

COPD: chronic obstructive pulmonary disease, FAR: the ratio of fibrinogen to albumin, WBC: white blood cell count, PT: prothombin time, APTT: activated partial thromboplastin time, MBP: mean blood pressure, SOFA: sepsis-related organ failure assessment, SAPSII: simplified acute physiology score II, MV: mechanical ventilation, RRT: renal replacement therapy, DIC: disseminated intravascular coagulation, AKI: acute kidney injury.

**Table 2:** FAR values based on time windows.

Time-windows	Variables	Total	No SA-AKI	SA-AKI	P-value
		n = 1587	n = 1203	n = 384	
<24 hours^*1^	Far, median (IQR)	81.5 (55.6, 128.8)	78.9 (55.3, 119.4)	91.0 (57.6, 154.8)	0.001
	Variables	n = 1070	n = 967	n = 103	
24-48 hours^*2^	Far, median (IQR)	81.1 (56.5, 125.9)	79.7 (56.1, 120.7)	103.9 (62.6, 185.9)	0.002
	Variables	n = 802	n = 763	n = 39	
48-72 hours^*3^	Far, median (IQR)	81.1 (56.9, 126.4)	80.5 (56.5, 124.5)	116.4 (63.3, 188.1)	0.011
	Variables	n = 805	n = 763	n = 42	
≥72 hours^*4^	Far, median (IQR)	82.9 (57.0, 127.1)	80.5 (56.5, 124.5)	116.8 (85.6, 180.9)	<0.001

*1: No SA-AKI after ICU day 1; SA-AKI onset within ICU day 1.

*2: No SA-AKI after ICU day 2; SA-AKI onset within ICU day 2.

*3: No SA-AKI after ICU day 3; SA-AKI onset within ICU day 3.

*4: No SA-AKI after ICU day 3; SA-AKI onset after ICU day 3.

### Logistic regression analysis

A univariable logistic regression analysis indicated risk factors associated with SA-AKI (Table 3). The association between FAR and SA‑AKI was further evaluated using multivariable logistic regression after adjusting for potential confounders (Table 4). Compared with the reference group, the crude odds ratio (OR) value for SA-AKI in the high-FAR group was 1.73 (95% CI 1.36–2.20, P < 0.001). After adjustment, the OR remained significant: 1.68 (95% CI 1.31–2.15, P < 0.001) in Model I; 1.57 (95% CI 1.22–2.03, P < 0.001) in Model II; and 1.55 (95% CI 1.11–2.18, P = 0.011) in Model III (Table 4). In a time-stratified Cox model (Table 5), the association between FAR and SA-AKI strengthened over time and became statistically significant after ICU day 3, with an adjusted HR of 5.17 (95% CI 1.81–14.72, P = 0.002).

**Table 3:** Risk factors for SA-AKI patients by univariate logistic analysis.

Variables	OR (95% CI)	P-value
Age, (per 10 yr)	1.070 (1.014-1.13)	0.014
Race		
Black	1.141 (0.769-1.694)	0.512
Other	1.053 (0.847-1.308)	0.642
Gender, (n%, Male)	0.995 (0.812-1.219)	0.964
Comorbidities, n (%)		
COPD	1.668 (1.117-2.493)	0.013
Hypertension	1.334 (1.092-1.629)	0.005
Diabetes	1.792 (1.417-2.266)	<0.001
Heart failure	2.571 (2.033-3.250)	<0.001
Laboratory parameters		
Far	1.004 (1.003-1.005)	<0.001
Fibrinogen, (mg/dL)	1.001 (1.001-1.002)	<0.001
Albumin, (g/dL)	0.628 (0.547-0.722)	<0.001
Creatinine, (mg/dL)	16.377 (11.826-22.679)	<0.001
Platelets, (K/uL)	0.999 (0.998-1.000)	0.024
WBC, (K/uL)	1.025 (1.014-1.036)	<0.001
PT	1.023 (1.015-1.031)	<0.001
APPT	1.005 (1.002-1.009)	0.005
Glucose, (mg/dL)	1.003 (1.002-1.004)	<0.001
MBP, (mmHg)	0.991 (0.986-0.996)	<0.001
Charlson score	1.167 (1.117-1.220)	<0.001
SOFA score	1.364 (1.298-1.432)	<0.001
SAPSII score	1.061 (1.053-1.070)	<0.001
Vasopressor use, n (%)	2.597 (2.077-3.247)	<0.001
MV use, n (%)	1.701 (1.308-2.213)	<0.001
DIC, n (%)	6.229 (3.483-11.141)	<0.001

COPD: chronic obstructive pulmonary disease, FAR: the ratio of fibrinogen to albumin, WBC: white blood cell count, PT: prothombin time, APTT: activated partial thromboplastin time, MBP: mean blood pressure, SOFA: sepsis-related organ failure assessment, SAPSII: simplified acute physiology scores II, MV: mechanical ventilation, DIC: disseminated intravascular coagulation.

**Table 4:** Multivariate logistic regression analysis of risk factors in SA-AKI patients.

Variables	N (% with AKI)	Crude model		Model I		Model II		Model III	
		OR (95% CI)	P-value	OR (95% CI)	P-value	OR (95% CI)	P-value	OR (95% CI)	P-value
FAR < 64.82	590 (28.6)	1 (Ref)		1 (Ref)		1 (Ref)		1 (Ref)	
64.82 ≤ FAR < 110.74	590 (26.6)	0.90 (0.70-1.17)	0.435	0.88 (0.68-1.13)	0.315	0.80 (0.61-1.04)	0.095	0.89 (0.64-1.25)	0.506
FAR ≥ 110.74	591 (40.9)	1.73 (1.36-2.20)	<0.001	1.68 (1.31-2.15)	<0.001	1.57 (1.22-2.03)	<0.001	1.55 (1.11-2.18)	0.011

Adjusted covariates:

Crude model: adjusted for no factors.

Model I: adjusted for age, gender, race.

Model II: adjusted for Model I and comorbidities (COPD, hypertension, diabetes, heart failure).

Model III: adjusted for Model II, Vasopressor use, MV, DIC, SOFA score, SAPSII score, Creatinine, PT, APTT, Platelets, WBC and MBP.

**Table 5:** Multivariate Cox regression analysis of risk factors in SA-AKI patients.

Time-windows	N (% with AKI)	FAR < 64.82	64.82 ≤ FAR < 110.74		FAR ≥ 110.74	
		HR (95% CI)	HR (95% CI)	P-value	HR (95% CI)	P-value
<24 hours^*1^	1587 (24.2)	1(Ref)	1.03 (0.79-1.35)	0.833	1.2 (0.93-1.56)	0.166
24-48 hours^*2^	1070 (9.6)	1(Ref)	0.92 (0.52-1.63)	0.772	1.63 (0.97-2.74)	0.066
48-72 hours^*3^	802 (4.9)	1(Ref)	0.61 (0.21-1.72)	0.348	2.15 (0.95-4.90)	0.067
≥72 hours^*4^	805 (5.2)	1(Ref)	1.99 (0.65-6.13)	0.23	5.17 (1.81-14.72)	0.002

*1: No SA-AKI after ICU day 1; SA-AKI onset within ICU day 1. *2: No SA-AKI after ICU day 2; SA-AKI onset within ICU day 2. *3: No SA-AKI after ICU day 3; SA-AKI onset within ICU day 3. *4: No SA-AKI after ICU day 3; SA-AKI onset after ICU day 3. The time-window Cox regression models adjusted for all variables: age, gender, race, COPD, hypertension, diabetes, heart failure, Vasopressor use, MV, DIC, SOFA score, SAPSII score, Creatinine, PT, APTT, Platelets, WBC and MBP.

### Sensitivity analysis

**Fig 3:**
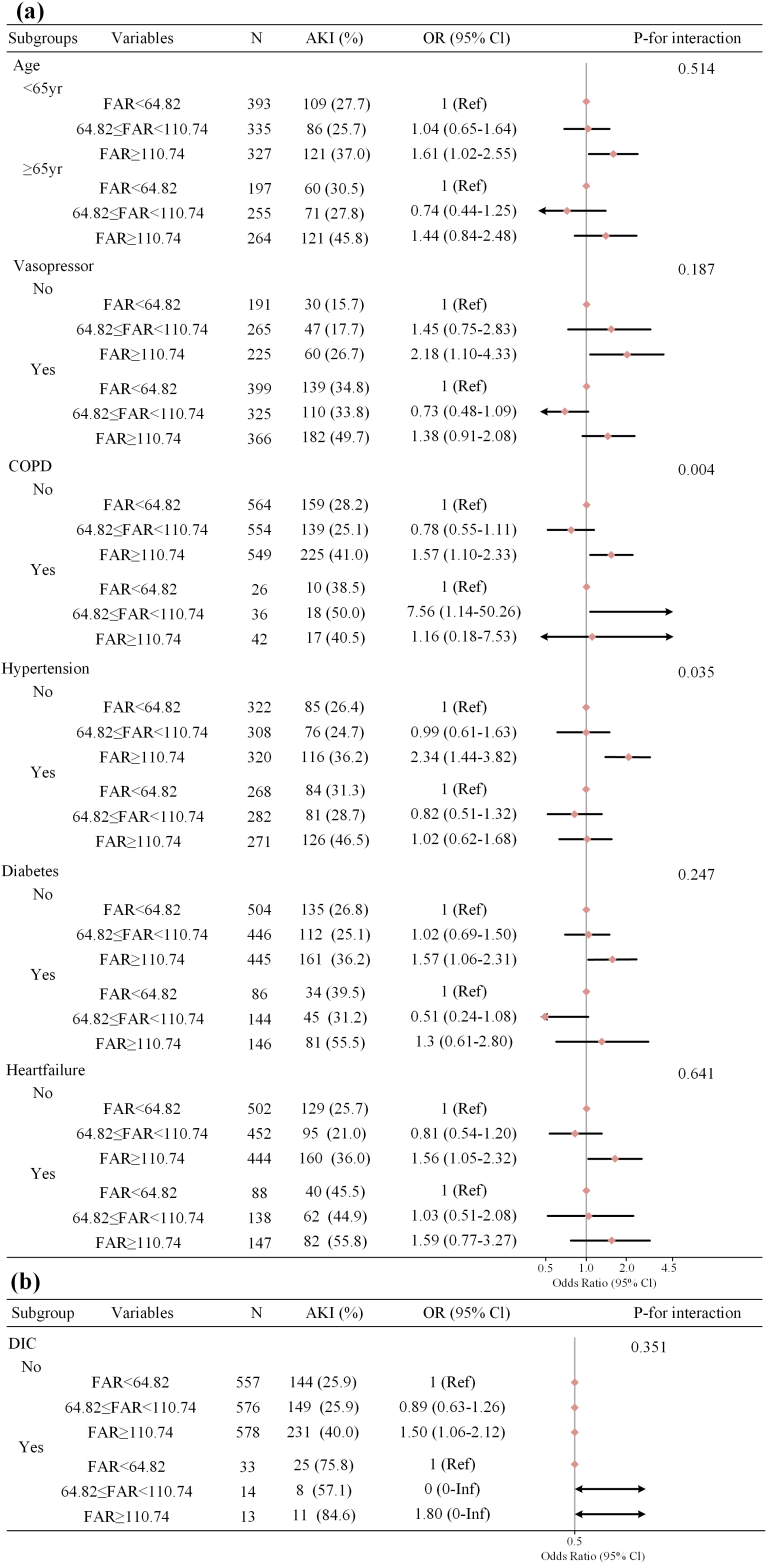
Subgroup analyses of the association between FAR and SA-AKI. Each stratification adjusted for the factors (gender, race, age, COPD, hypertension, diabetes, heart failure, Vasopressor use, MV, DIC, SOFA score, SAPSII score, Creatinine, PT, APTT, Platelets, WBC and MBP) except the stratification factor itself. COPD = chronic obstructive pulmonary disease, MV = mechanical ventilation, DIC = disseminated intravascular coagulation, SOFA = sepsis-related organ failure assessment, SAPSII = simplified acute physiology score II, PT = prothombin time, APTT = activated partial thromboplastin time, WBC = white blood cell count, MBP = mean blood pressure.

The results of subgroup analyses, which were stratified by age, vasopressor use, DIC complication, diabetes and heart failure (Fig 3), were similar to the main finding (Table 4), and there were no interaction between variables listed above. However, the interactions between other subgroups stratified by COPD and hypertension were observed (Fig 3a). After excluding patients with COPD or with hypertension, the multivariate logistic and Cox regression analyses were conducted further. As shown in Table 6 and Table 7, the adjusted OR in Model III was 2.31 (95% CI 1.40–3.82, P = 0.001), and the adjusted HR in the ≥ 72‑hour group was 8.57 (95% CI 1.88–38.94, P = 0.005), which aligns well with the association reported in Table 4 and Table 5, respectively.

**Table 6:** Multivariate logistic regression analysis of risk factors for SA-AKI in patients without COPD and hypertension.

Variables	N (% with AKI)	Crude model		Model I		Model II		Model III	
		OR (95% CI)	P-value	OR (95% CI)	P-value	OR (95% CI)	P-value	OR (95% CI)	P-value
FAR < 64.82	310 (25.8)	1(Ref)		1(Ref)		1(Ref)		1(Ref)	
64.82 ≤ FAR < 110.74	287 (23.3)	0.88 (0.60-1.27)	0.486	0.87 (0.60-1.27)	0.474	0.78 (0.53-1.16)	0.219	0.83 (0.49-1.40)	0.482
FAR ≥ 110.74	301 (35.9)	1.61 (1.14-2.28)	0.007	1.6 (1.12-2.28)	0.010	1.57 (1.09-2.25)	0.016	2.31 (1.40-3.82)	0.001

Adjusted covariates:

Crude model: adjusted for no factors.

Model I: adjusted for age, gender and race.

Model II: adjusted for Model I, diabetes and heart failure.

Model III: adjusted for Model II, Vasopressor use, MV, DIC, SOFA score, SAPSII score, Creatinine, PT, APTT, Platelets, WBC and MBP.

**Table 7:** Multivariate Cox regression analysis of risk factors for SA-AKI in patients without COPD and hypertension.

Time-windows	N (% with AKI)	FAR < 64.82	64.82 ≤ FAR < 110.74		FAR ≥ 110.74	
		HR (95% CI)	HR (95% CI)	P-value	HR (95% CI)	P-value
<24 hours^*1^	812 (20.8)	1(Ref)	0.9 (0.60-1.35)	0.603	1.12 (0.76-1.66)	0.553
24-48 hours^*2^	568 (8.3)	1(Ref)	0.6 (0.25-1.49)	0.272	1.75 (0.83-3.69)	0.144
48-72 hours^*3^	432 (4.2)	1(Ref)	0.14 (0.01-1.42)	0.095	3.55 (0.91-13.84)	0.068
≥72 hours^*4^	435 (4.8)	1(Ref)	3.94 (0.81-19.23)	0.090	8.57 (1.88-38.94)	0.005

*1: No SA-AKI after ICU day 1; SA-AKI onset within ICU day 1. *2: No SA-AKI after ICU day 2; SA-AKI onset within ICU day 2. *3: No SA-AKI after ICU day 3; SA-AKI onset within ICU day 3. *4: No SA-AKI after ICU day 3; SA-AKI onset after ICU day 3. The time-window Cox regression models adjusted for all variables: age, gender, race, diabetes, heart failure, Vasopressor use, MV, DIC, SOFA score, SAPSII score, Creatinine, PT, APTT, Platelets, WBC and MBP.

**Fig 4:**
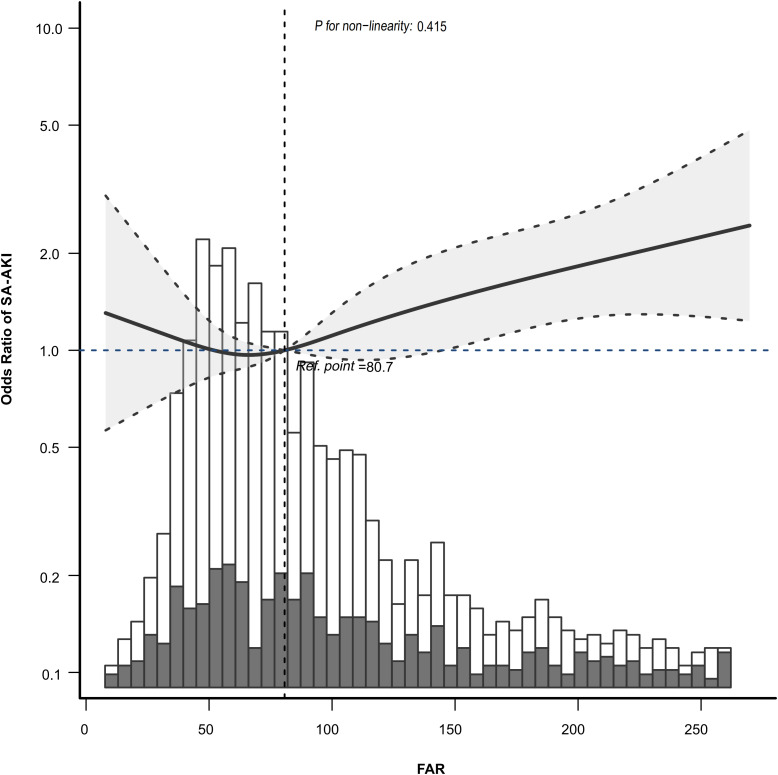
RCS curve fitting of relationship between FAR with SA-AKI. The solid line and dashed line represented the estimated values and their corresponding 95% confidence intervals. Only 95% of the data were displayed. Adjusted for all factors (age, gender, race, COPD, hypertension, diabetes, heart failure,Vasopressor use, MV, DIC, SOFA score, SAPSII score, Creatinine, PT, APTT, Platelets, WBC and MBP).

The relationship between FAR and SA-AKI was further fitted using RCS curve. It was shown that RCS analysis indicated a linear relationship between FAR and SA-AKI (P for non-linearity: 0.415) (Fig 4), consistent with the results presented in Table 4.

### Prognostic value of variables

**Fig 5:**
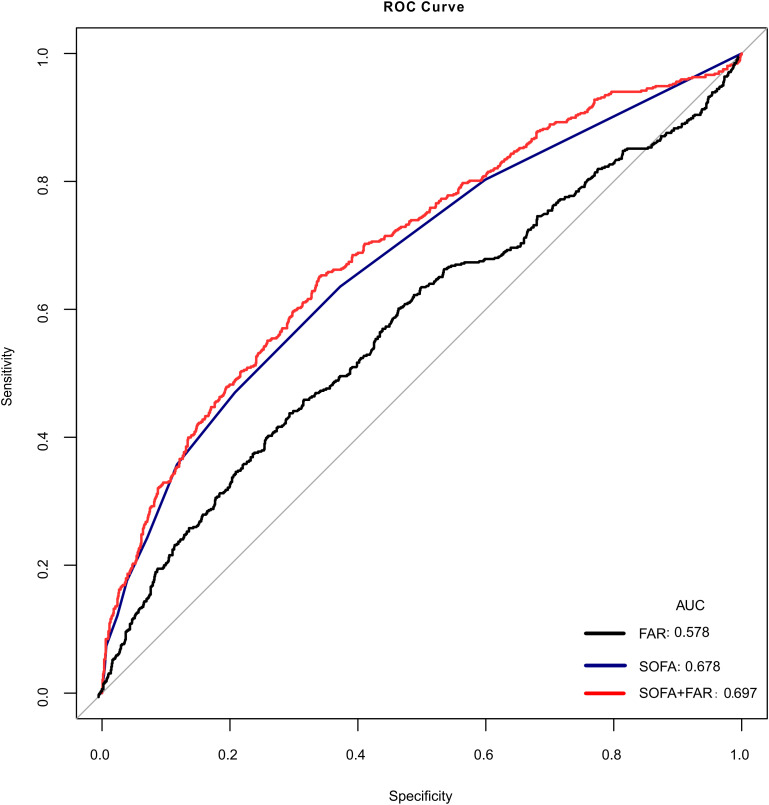
The ROCs for FAR, SOFA, and FAR combined with SOFA in predicting the incidence of SA-AKI. ROC: receiver operating characteristic curve, AUC: area under the curve, FAR: the ratio of fibrinogen to albumin, SOFA: sepsis-related organ failure assessment, SA-AKI: sepsis-associated acute kidney injury.

We further evaluated the predictive performance of FAR and other clinical marker (Table 8). The sensitivity, specificity, positive predictive value (PPV), and negative predictive value (NPV) of FAR were 0.444, 0.702, 0.412 and 0.728, respectively. In the ROC analysis, FAR combined with SOFA slightly outperformed SOFA alone (AUC 0.697 vs. 0.678; P = 0.004) (Fig 5).

**Table 8:** Comparison of prognostic FAR and other clinical markers.

Variables	ROC Areas (AUCs)	P-value	95% Confidence Interval		Sensitivity	Specificity	PPV	NPV
			95% CI Lower	95% CI Upper				
FAR	0.578	<0.001^*1^	0.548	0.607	0.444	0.702	0.412	0.728
SOFA	0.678	0.004^*2^	0.651	0.704	0.636	0.628	0.446	0.785
FAR+SOFA	0.697	——	0.670	0.724	0.651	0.660	0.475	0.800

*1: The comparison of the predictive ability for SA-AKI between FAR and FAR combined with SOFA, *2: The comparison of the predictive ability for SA-AKI between SOFA and FAR combined with SOFA. ROC: receiver operating characteristic curve, AUCs: areas under the curve, CI: confidence interval, PPV: positive predictive value, NPV: negative predictive value, FAR: the ratio of fibrinogen to albumin, SOFA: sepsis-related organ failure assessment.

## Discussion

The main finding of the present study is that an elevated FAR level is associated with the increased incidence of SA-AKI. Patients with elevated FAR levels, particularly those in the highest tertile, exhibited a significantly higher rate of SA-AKI. However, the association was significant only when SA-AKI occurred after ICU day 3 among patients with sepsis who had neither COPD nor hypertension.

The detrimental inflammatory cascade characteristic of sepsis seems to be an essential player in the pathogenesis of AKI [14]. Fibrinogen as a key regulator of inflammation in disease [15], correlates with excessive inflammation and disease severity [16]. Additionally, fibrinogen, as a key component in hemostasis and coagulation, induces platelet aggregation via glycoprotein IIb-IIIa [17], disrupting renal hemodynamics and promoting sustained hypoxic renal injury and inflammation [18]. Sustained low albumin level reflects ongoing inflammation and is linked to cardiovascular disease, rather than malnutrition [19,20], and adequate levels of serum albumin are also essential for maintaining oncotic pressure and ensuring perfusion [21]. Both of which above are risk factors for AKI and may contribute to sepsis-induced AKI [22]. FAR, as a novel inflammatory biomarker [23], has shown promise in various medical conditions, including cancer mortality [24], thromboembolic complications [25] and in-hospital mortality among critically patients with AKI [26]. However, its role in SA-AKI had not been thoroughly investigated until now. In our study, we revealed a positive association between FAR and SA-AKI, independent of other well established risk factors (Table 4), and the RCS curve fitting also showed an adjusted, continuous association between FAR and SA-AKI (Fig 4). There was a positive correlation between fibrinogen and AKI [27], while negative correlation between albumin and AKI [28]. FAR incorporates both of these opposing factors, which amplified the distinction. FAR may be more valuable than fibrinogen and albumin in assessing the SA-AKI in sepsis patients. Consequently, FAR combined with SOFA may offer greater clinical value than SOFA alone in assessing SA-AKI risk in sepsis patients (Table 8, Fig 5). Therefore, early risk assessment through FAR measurement may enable clinicians to implement timely interventions and preventive strategies, ultimately reducing the occurrence and severity of SA-AKI in sepsis patients.

The temporal association between FAR and SA-AKI reached significant only when SA-AKI occurred after ICU day 3 (Table 5). Across different time windows, the ≥ 72-hour SA-AKI group had the highest FAR, and the FAR difference in the ≥ 72-hour window was the largest among all time windows (Table 2, Fig 2), which made the association between FAR and SA-AKI in this time window more statistically significant than in the other groups. This temporal association (Table 5) were still consistent with the main finding (Table 4), indicating that higher FAR was associated with a greater risk of SA-AKI. The ≥ 72-hour group had milder illness than the other groups (S3 Table, S4 Table). Among patients with milder sepsis, fibrinogen levels were higher, consistent with the study by Chi Yao et al. [29]. In addition, the ≥ 72-hour group had a lower incidence of DIC, which may further help explain the higher FAR in this group relative to the other time windows. Early in sepsis, hemodynamic instability can further exacerbate kidney injury [30], especially in the sicker cohort, which might obscure the individual effect of the FAR. In contrast, after 72 hours, persistent inflammatory infiltration and microthrombosis formation lead to microcirculatory disturbances and sustained kidney damage [31]. Therefore, the FAR might better reflect the cumulative inflammatory damage rather than acute changes.

Subgroup analyses suggested interactions in COPD and hypertension groups (Fig 3). COPD entails chronic systemic inflammation and hypoxia, leading to endothelial and microcirculatory dysfunction [32,33], and hypertension promotes inflammation and fibrosis via renin–angiotensin–aldosterone system overactivation and microvascular remodeling [34]. Both of which can alter FAR and weaken its association with SA-AKI. Additionally, a markedly smaller COPD sample may have reduced the precision of effect-size estimation. After excluding patients with COPD and hypertension, the findings (Table 6, Table 7) remained robust.

There are some unavoidable limitations in our study. Firstly, this single-center retrospective study was prone to selection bias, and association does not imply causation or prediction performance. Secondly, many patients lacked the first FAR values (Fig 1). Our study results were more applicable to the sepsis subgroup with an indication for FAR testing. Future studies should expand the sample to further investigate the association between FAR and SA-AKI. Thirdly, FAR correlates with an acute-phase response, high FAR may have been a result of SA-AKI. Fourthly,. COPD and hypertension interfered with the FAR–SA-AKI association, limiting generalizability in these subgroups. Finally, CKD patients were excluded because MIMIC could not distinguish CKD with AKI from CKD alone. Therefore, future studies should validate this association with a prospective design.

## Conclusions

An elevated FAR level was associated with an increased incidence of SA-AKI in patients without COPD and hypertension. However, this association reached statistical significance only when SA-AKI occurred after ICU day 3. Further research is needed to explore this association. And the predictive ability of FAR for SA-AKI still be required further exploration.

## Supporting information

S1 Table Data on albumin and fibrinogen values in ICU patients. (DOCX)

S2 Table Data on missing values in the study. (DOCX)

S3 Table Baseline characteristics of SA-AKI patients across different time windows. (DOCX)

S4 Table Baseline characteristics of participants across different time windows. (DOCX)

## Abbreviations

SA-AKI: Sepsis-associated acute kidney injury
KDIGO: Kidney Disease: Improving Global Outcomes
FAR: Fibrinogen-to-albumin ratio
STROBE: The Strengthening the Reporting of Observational Studies in Epidemiology
RECORD: Reporting of studies Conducted using Observational Routinely-collected Data
MIMIC: Medical Information Mart for Intensive Care
ICU: Intensive care unit
CKD: chronic kidney disease
COPD: Chronic obstructive pulmonary disease
WBC: White blood cell count
PT: Prothrombin time
APTT: Activated partial thromboplastin time
MBP: Mean blood pressure
SOFA: Sepsis-related organ failure assessment
SAPSII: Simplified acute physiology score II
MV: Mechanical ventilation
RRT: Renal replacement therapy
DIC: Disseminated intravascular coagulation
AKI: Acute kidney injury
SD: Standard deviation
IQR: Interquartile range
VIF: Variance inflation factor
RCS: Restricted cubic spline
ROC: receiver operating characteristic curve
AUC: area under the curve
OR: Odds ratio
CIs: Confidence intervals
PPV: positive predictive value
NPV: negative predictive value

## References

[1] Manrique-Caballero CL, Del Rio-Pertuz G, Gomez H. Sepsis-associated acute kidney injury. Crit Care Clin. 2021;37(2):279–301. doi: 10.1016/j.ccc.2020.11.010 33752856 PMC7995616

[2] Peerapornratana S, Manrique-Caballero CL, Gómez H, Kellum JA. Acute kidney injury from sepsis: Current concepts, epidemiology, pathophysiology, prevention and treatment. Kidney Int. 2019;96(5):1083–99. doi: 10.1016/j.kint.2019.05.026 31443997 PMC6920048

[3] Zarbock A, Nadim MK, Pickkers P, Gomez H, Bell S, Joannidis M, et al. Sepsis-associated acute kidney injury: Consensus report of the 28th Acute Disease Quality Initiative workgroup. Nat Rev Nephrol. 2023;19(6):401–17. doi: 10.1038/s41581-023-00683-3 36823168

[4] Sun D-W, An L, Lv G-Y. Albumin-fibrinogen ratio and fibrinogen-prealbumin ratio as promising prognostic markers for cancers: An updated meta-analysis. World J Surg Oncol. 2020;18(1):9. doi: 10.1186/s12957-020-1786-2 31931816 PMC6958612

[5] Binti NN, Ferdausi N, Anik MEK, Islam LN. Association of albumin, fibrinogen, and modified proteins with acute coronary syndrome. PLoS One. 2022;17(7):e0271882. doi: 10.1371/journal.pone.0271882 35881574 PMC9321412

[6] Tai H, Zhu Z, Mei H, Sun W, Zhang W. Albumin-to-fibrinogen ratio independently predicts 28-day mortality in patients with peritonitis-induced sepsis. Mediators of Inflammation. 2020;2020:1–7. doi: 10.1155/2020/7280708 PMC7225846 32454793

[7] Zou Y, Zhu Z, Zhou J, Wu X, Li H, Ning X, et al. Fibrinogen/Albumin ratio: A more powerful prognostic index for patients with end-stage renal disease. Eur J Clin Invest. 2020;:e13266. doi: 10.1111/eci.13266 32379901

[8] von Elm E, Altman DG, Egger M, Pocock SJ, Gøtzsche PC, Vandenbroucke JP, et al. The Strengthening the Reporting of Observational Studies in Epidemiology (STROBE) Statement: guidelines for reporting observational studies. Int J Surg. 2014;12(12):1495–9. doi: 10.1016/j.ijsu.2014.07.013 25046131

[9] Chen MY, Langan S, Benchimol EI. Routinely collected electronic health data and STI research: RECORD extension to the STROBE guidelines. Sex Transm Infect. 2016;92(1):2–3. doi: 10.1136/sextrans-2015-052360 26668088

[10] Johnson AEW, Bulgarelli L, Shen L, Gayles A, Shammout A, Horng S, et al. MIMIC-IV, a freely accessible electronic health record dataset. Sci Data. 2023;10(1):1. doi: 10.1038/s41597-022-01899-x 36596836 PMC9810617

[11] Johnson A, Bulgarelli L, Pollard T, Horng S, Celi LA, Mark R. MIMIC-IV (version 2.0). PhysioNet. 10.13026/7vcr-e114. 2022.

[12] Singer M, Deutschman CS, Seymour CW, Shankar-Hari M, Annane D, Bauer M, et al. The third international consensus definitions for sepsis and septic shock (Sepsis-3). JAMA. 2016;315(8):801–10. doi: 10.1001/jama.2016.0287 26903338 PMC4968574

[13] Chang Y-M, Chou Y-T, Kan W-C, Shiao C-C. Sepsis and acute kidney injury: A review focusing on the bidirectional interplay. Int J Mol Sci. 2022;23(16):9159. doi: 10.3390/ijms23169159 36012420 PMC9408949

[14] Morrell ED, Kellum JA, Pastor-Soler NM, Hallows KR. Septic acute kidney injury: Molecular mechanisms and the importance of stratification and targeting therapy. Crit Care. 2014;18(5):501. doi: 10.1186/s13054-014-0501-5 25575158 PMC4729166

[15] Davalos D, Akassoglou K. Fibrinogen as a key regulator of inflammation in disease. Semin Immunopathol. 2012;34(1):43–62. doi: 10.1007/s00281-011-0290-8 22037947

[16] Sui J, Noubouossie DF, Gandotra S, Cao L. Elevated plasma fibrinogen is associated with excessive inflammation and disease severity in COVID-19 patients. Front Cell Infect Microbiol. 2021;11:734005. doi: 10.3389/fcimb.2021.734005 34414135 PMC8369350

[17] Bennett JS. Platelet-fibrinogen interactions. Ann N Y Acad Sci. 2001;936:340–54. doi: 10.1111/j.1749-6632.2001.tb03521.x 11460491

[18] Jansen MPB, Florquin S, Roelofs JJTH. The role of platelets in acute kidney injury. Nat Rev Nephrol. 2018;14(7):457–71. doi: 10.1038/s41581-018-0015-5 29760447

[19] Manolis AA, Manolis TA, Melita H, Mikhailidis DP, Manolis AS. Low serum albumin: A neglected predictor in patients with cardiovascular disease. Eur J Intern Med. 2022;102:24–39. doi: 10.1016/j.ejim.2022.05.004 35537999

[20] Soeters PB, Wolfe RR, Shenkin A. Hypoalbuminemia: Pathogenesis and clinical significance. JPEN J Parenter Enteral Nutr. 2019;43(2):181–93. doi: 10.1002/jpen.1451 30288759 PMC7379941

[21] Hassan K, Kristal B, Hassan F, Abo Saleh S, Michelis R. The impact of oxidized serum albumin on the oncotic pressure and hydration status of peritoneal dialysis patients. Ther Clin Risk Manag. 2016;12:463–9. doi: 10.2147/TCRM.S102311 27069365 PMC4818040

[22] Chen L, Wu X, Qin H, Zhu H. The PCT to albumin ratio predicts mortality in patients with acute kidney injury caused by abdominal infection-evoked sepsis. Front Nutr. 2021;8:584461. doi: 10.3389/fnut.2021.584461 34141715 PMC8203818

[23] Huang R, Dai Q, Chang L, Wang Z, Chen J, Gu R, et al. The association between fibrinogen-to-albumin ratio (FAR) and adverse prognosis in patients with acute decompensated heart failure at different glucose metabolic states. Cardiovasc Diabetol. 2022;21(1):241. doi: 10.1186/s12933-022-01662-x 36371183 PMC9655790

[24] Wen Y, Yang J, Han X. Fibrinogen-to-albumin ratio is associated with all-cause mortality in cancer patients. Int J Gen Med. 2021;14:4867–75. doi: 10.2147/IJGM.S322735 34475778 PMC8407668

[25] Roth S, Jansen C, M’Pembele R, Stroda A, Boeken U, Akhyari P, et al. Fibrinogen-Albumin-Ratio is an independent predictor of thromboembolic complications in patients undergoing VA-ECMO. Sci Rep. 2021;11(1):16648. doi: 10.1038/s41598-021-95689-x 34404824 PMC8371004

[26] Xia W, Li C, Yao X, Chen Y, Zhang Y, Hu H. Prognostic value of fibrinogen to albumin ratios among critically ill patients with acute kidney injury. Intern Emerg Med. 2022;17(4):1023–31. doi: 10.1007/s11739-021-02898-3 34850361 PMC9135817

[27] Yang JJ, Lei WH, Hu P, Wu BB, Chen JX, Ni YM, et al. Preoperative serum fibrinogen is associated with acute kidney injury after cardiac valve replacement surgery. Sci Rep. 2020;10(1):6403. doi: 10.1038/s41598-020-63522-6 32286477 PMC7156756

[28] Nada A, Askenazi D, Kupferman JC, Mhanna M, Mahan JD, Boohaker L, et al. Low albumin levels are independently associated with neonatal acute kidney injury: A report from AWAKEN Study Group. Pediatr Nephrol. 2022;37(7):1675–86. doi: 10.1007/s00467-021-05295-2 34657971 PMC9986677

[29] Yao C, Zhang G, Zhang N, Li R, Sun S, Zhang L, et al. Fibrinogen Is associated with prognosis of critically ill patients with sepsis: A study based on cox regression and propensity score matching. Mediators Inflamm. 2023;2023:7312822. doi: 10.1155/2023/7312822 36994229 PMC10042635

[30] Badr KF, Ichikawa I. Prerenal failure: A deleterious shift from renal compensation to decompensation. N Engl J Med. 1988;319(10):623–9. doi: 10.1056/NEJM198809083191007 3045546

[31] Kuwabara S, Goggins E, Okusa MD. The pathophysiology of sepsis-associated AKI. Clin J Am Soc Nephrol. 2022;17(7):1050–69. doi: 10.2215/CJN.00850122 35764395 PMC9269625

[32] Aoshiba K, Nagai A. Differences in airway remodeling between asthma and chronic obstructive pulmonary disease. Clin Rev Allergy Immunol. 2004;27(1):35–43. doi: 10.1385/CRIAI:27:1:035 15347849

[33] Sutherland ER, Martin RJ. Airway inflammation in chronic obstructive pulmonary disease: Comparisons with asthma. J Allergy Clin Immunol. 2003;112(5):819–27; quiz 828. doi: 10.1016/S0091 14610463

[34] Zhang Z, Zhao L, Zhou X, Meng X, Zhou X. Role of inflammation, immunity, and oxidative stress in hypertension: New insights and potential therapeutic targets. Front Immunol. 2023;13:1098725. doi: 10.3389/fimmu.2022.1098725 36703963 PMC9871625

