# Supplementary material for: Association of fibrinogen to albumin ratio with sepsis-associated acute kidney injury: A retrospective cohort study based on the MIMIC-IV database
Source: PLoS One. 2026 Mar 6;21(3):e0343549. doi: 10.1371/journal.pone.0343549 (PMC12965584; doi:10.1371/journal.pone.0343549)
Supplement: S1 Table — (DOCX) [file pone.0343549.s001.docx]

**Table S1. Data on albumin and fibrinogen values in ICU patients**

|  | The number of patients | The number of patients with missing initial albumin values (percentage of total patients) | The number of patients with missing initial fibrinogen values (percentage of total patients) | The number of patients with missing initial albumin or fibrinogen values (percentage of total patients) | The number of patients with both initial albumin and fibrinogen values (percentage of total patients) |
| --- | --- | --- | --- | --- | --- |
| All ICU patients diagnosed with non-sepsis | 42044 | 27924 (66.4%) | 33399 (79.4%) | 39209 (93.3%) | 2835 (6.7%) |
| All ICU patients diagnosed with sepsis | 34899 | 14504 (41.6%) | 21082 (60.4%) | 26394 (75.6%) | 8505 (24.4%) |
| All ICU patients diagnosed with sepsis for the first time during each hospitalization period | 33019 | 13805 (41.8%) | 19996 (60.6%) | 25126 (76.1%) | 7893 (23.9%) |
| ICU sepsis patients in this study | 1771 | 0 (0%) | 0 (0%) | 0 (0%) | 1771 (100%) |
