# Supplementary material for: Association of fibrinogen to albumin ratio with sepsis-associated acute kidney injury: A retrospective cohort study based on the MIMIC-IV database
Source: PLoS One. 2026 Mar 6;21(3):e0343549. doi: 10.1371/journal.pone.0343549 (PMC12965584; doi:10.1371/journal.pone.0343549)
Supplement: S2 Table — (DOCX) [file pone.0343549.s002.docx]

| Variables | The number of missing data |
| --- | --- |
| Age | 0 |
| Race | 0 |
| Gender | 0 |
| COPD | 0 |
| Hypertension | 0 |
| Diabetes | 0 |
| Heart failure | 0 |
| Far | 0 |
| Fibrinogen | 0 |
| Albumin | 0 |
| Creatinine | 0 |
| Platelets | 0 |
| WBC | 0 |
| PT | 2 |
| APTT | 3 |
| Glucose | 0 |
| MBP | 9 |
| Charlson score | 0 |
| SOFA score | 0 |
| SAPSII score | 0 |
| Vasopressor use | 0 |
| MV use | 0 |
| RRT | 0 |
| Hospital LOS | 0 |
| ICU LOS | 0 |
| ICU Mortality | 0 |
| Hospital Mortality | 0 |
| 30-day Mortality | 0 |
| DIC | 0 |
| AKI | 0 |
| AKI stage | 0 |

**Table S2. Data on missing values in the study**

COPD: chronic obstructive pulmonary disease, FAR: the ratio of fibrinogen to albumin, WBC: white blood cell count, PT: Prothombin time, APTT: activated partial thromboplastin time, MBP: mean blood pressure, SOFA: sepsis-related organ failure assessment, SAPSII: simplified acute physiology score II, MV: mechanical ventilation, RRT: renal replacement therapy, DIC: disseminated intravascular coagulation, AKI: acute kidney injury.
