# Supplementary material for: Association of fibrinogen to albumin ratio with sepsis-associated acute kidney injury: A retrospective cohort study based on the MIMIC-IV database
Source: PLoS One. 2026 Mar 6;21(3):e0343549. doi: 10.1371/journal.pone.0343549 (PMC12965584; doi:10.1371/journal.pone.0343549)
Supplement: S3 Table — (DOCX) [file pone.0343549.s003.docx]

Table S3. Baseline characteristics of SA-AKI patients across different time windows

| Variables | Total | <24 hour^*1^ | 24-48 hours^*2^ | 48-72 hours^*3^ | ≥72 hours^*4^ | P-value |
| --- | --- | --- | --- | --- | --- | --- |
|  | (n=568) | (n=384) | (n=103) | (n=39) | (n=42) |  |
| Age (yr),  median (IQR) | 62.5  (47.2, 74.9) | 63.7  (48.2, 75.7) | 62.0  (43.5, 74.9) | 63.7  (58.9, 71.7) | 54.5  (39.0, 68.9) | 0.146 |
| Race, n (%) |  |  |  |  |  | 0.111 |
| White | 340 (59.9) | 225 (58.6) | 57 (55.3) | 30 (76.9) | 28 (66.7) |  |
| Black | 42 ( 7.4) | 25 (6.5) | 13 (12.6) | 2 (5.1) | 2 (4.8) |  |
| Other | 186 (32.7) | 134 (34.9) | 33 (32) | 7 (17.9) | 12 (28.6) |  |
| Gender, n (%) |  |  |  |  |  | 0.033 |
| Female | 232 (40.8) | 149 (38.8) | 55 (53.4) | 14 (35.9) | 14 (33.3) |  |
| Male | 336 (59.2) | 235 (61.2) | 48 (46.6) | 25 (64.1) | 28 (66.7) |  |
| Comorbidities, n (%) |  |  |  |  |  |  |
| Comorbidities | 422 (74.3) | 289 (75.3) | 73 (70.9) | 30 (76.9) | 30 (71.4) | 0.767 |
| COPD | 45 ( 7.9) | 35 (9.1) | 6 (5.8) | 3 (7.7) | 1 (2.4) | 0.420 |
| Hypertension | 291 (51.2) | 197 (51.3) | 53 (51.5) | 20 (51.3) | 21 (50.0) | 0.999 |
| Diabetes | 160 (28.2) | 111 (28.9) | 24 (23.3) | 13 (33.3) | 12 (28.6) | 0.609 |
| Heart failure | 184 (32.4) | 113 (29.4) | 35 (34) | 17 (43.6) | 19 (45.2) | 0.070 |
| Laboratory parameters |  |  |  |  |  |  |
| Far,  median (IQR) | 96.6  (60.7, 171.9) | 91.0  (57.6, 154.8) | 103.9  (62.6, 185.9) | 116.4  (63.3, 188.1) | 116.8  (85.6, 180.9) | 0.041 |
| Fibrinogen (mg/dL) ,  median (IQR) | 298.5  (192.8, 466.5) | 285.5  (171.8, 438.5) | 315.0  (215.0, 520.5) | 351.0  (218.5, 563.5) | 404.5  (297.8, 536.5) | <0.001 |
| Albumin (g/dL),  mean (SD) | 3.1 ± 0.7 | 3.0 ± 0.8 | 3.1 ± 0.6 | 3.2 ± 0.7 | 3.2 ± 0.6 | 0.112 |
| Creatinine (mg/dL) ,  median (IQR) | 1.5  (1.1, 2.1) | 1.5  (1.1, 2.3) | 1.4  (1.1, 1.8) | 1.5  (1.1, 1.8) | 1.4  (1.2, 1.8) | 0.123 |
| Platelets (K/uL) ,  median (IQR) | 185.5  (112.0, 266.0) | 176.5  (108.0, 258.0) | 189.0  (115.5, 274.0) | 189.0  (123.5, 268.0) | 213.0  (162.5, 276.2) | 0.069 |
| WBC (K/uL),  median (IQR) | 13.0 (8.4, 19.7) | 13.3 (8.5, 19.9) | 12.3 (8.3, 18.8) | 13.0 (7.5, 18.0) | 11.9 (8.4, 18.9) | 0.561 |
| PT,  median (IQR) | 15.8  (13.0, 21.9) | 15.9  (13.0, 23.0) | 15.6  (12.8, 19.6) | 16.0  (13.1, 17.9) | 15.4  (13.7, 20.4) | 0.828 |
| APTT,  median (IQR) | 32.9  (28.1, 44.4) | 33.4  (28.5, 47.1) | 31.2  (27.4, 41.2) | 33.4  (28.4, 37.8) | 29.9  (26.8, 37.6) | 0.047 |
| Glucose (mg/dL),  median (IQR) | 148.0  (112.8, 195.0) | 150.0  (114.0, 200.0) | 137.0  (112.0, 179.5) | 158.0  (121.5, 202.5) | 135.5  (105.0, 180.5) | 0.341 |
| MBP (mmHg), mean (SD) | 82.1 ± 21.1 | 82.2 ± 21.1 | 83.6 ± 23.8 | 79.7 ± 14.5 | 80.1 ± 19.4 | 0.705 |
| Charlson score,  median (IQR) | 5.0  (3.0, 6.0) | 5.0  (3.0, 6.0) | 5.0  (2.0, 6.0) | 5.0  (4.0, 6.0) | 3.5  (2.0, 5.0) | 0.021 |
| SOFA score,  median (IQR) | 4.0  (3.0, 6.0) | 5.0  (3.0, 7.0) | 4.0  (2.5, 6.0) | 4.0  (2.0, 5.5) | 3.0  (2.0, 5.8) | <0.001 |
| SAPSII score,  median (IQR) | 46.0  (36.0, 56.0) | 49.5  (39.0, 60.0) | 39.0  (31.0, 49.5) | 39.0  (34.5, 48.0) | 33.0  (25.2, 42.5) | <0.001 |
| Vasopressor use, n (%) | 431 (75.9) | 300 (78.1) | 72 (69.9) | 31 (79.5) | 28 (66.7) | 0.152 |
| MV use, n (%) | 480 (84.5) | 326 (84.9) | 82 (79.6) | 36 (92.3) | 36 (85.7) | 0.285 |
| Clinical outcomes |  |  |  |  |  |  |
| RRT, n (%) | 137 (24.1) | 113 (29.4) | 16 (15.5) | 6 (15.4) | 2 (4.8) | <0.001 |
| Hospital LOS (days),  median (IQR) | 11.9  (6.6, 21.1) | 10.3  (5.2, 20.6) | 13.8  (7.7, 21.0) | 15.8  (10.6, 24.5) | 14.8  (11.5, 26.3) | <0.001 |
| ICU LOS (days),  median (IQR) | 6.4  (3.0, 12.4) | 5.7  (2.6, 11.2) | 6.7  (3.4, 12.7) | 11.2  (5.7, 19.1) | 9.8  (6.7, 15.7) | <0.001 |
| ICU Mortality, n (%) | 162 (28.5) | 126 (32.8) | 21 (20.4) | 11 (28.2) | 4 (9.5) | 0.003 |
| Hospital Mortality, n (%) | 178 (31.3) | 140 (36.5) | 23 (22.3) | 10 (25.6) | 5 (11.9) | <0.001 |
| 30-day Mortality, n (%) | 203 (35.7) | 157 (40.9) | 28 (27.2) | 11 (28.2) | 7 (16.7) | 0.002 |
| DIC, n (%) | 44 ( 7.7) | 36 (9.4) | 4 (3.9) | 3 (7.7) | 1 (2.4) | 0.175 |
| AKI stage, n (%) |  |  |  |  |  | <0.001 |
| 1 | 429 (75.5) | 267 (69.5) | 92 (89.3) | 32 (82.1) | 38 (90.5) |  |
| 2 | 130 (22.9) | 110 (28.6) | 10 (9.7) | 6 (15.4) | 4 (9.5) |  |
| 3 | 9 (1.6) | 7 (1.8) | 1 (1.0) | 1 (2.6) | 0 (0.0) |  |

*1: SA-AKI onset within ICU day 1. *2: SA-AKI onset within ICU day 2. *3: SA-AKI onset within ICU day 3. *4: SA-AKI onset after ICU day 3. Comorbidities: any of COPD, hypertension, diabetes and heart failure. COPD: chronic obstructive pulmonary disease, FAR: the ratio of fibrinogen to albumin, WBC: white blood cell count, PT: prothombin time, APTT: activated partial thromboplastin time, MBP: mean blood pressure, SOFA: sepsis-related organ failure assessment, SAPSII: simplified acute physiology score II, MV: mechanical ventilation, RRT: renal replacement therapy, DIC: disseminated intravascular coagulation, AKI: acute kidney injury.
