# Supplementary material for: Association of fibrinogen to albumin ratio with sepsis-associated acute kidney injury: A retrospective cohort study based on the MIMIC-IV database
Source: PLoS One. 2026 Mar 6;21(3):e0343549. doi: 10.1371/journal.pone.0343549 (PMC12965584; doi:10.1371/journal.pone.0343549)
Supplement: S4 Table — (DOCX) [file pone.0343549.s004.docx]

Table S4. Baseline characteristics of participants across different time windows

| Variables | Total | <24 hour^*1^ | 24-48 hours^*2^ | 48-72 hours^*3^ | ≥72 hours^*4^ | P-value |
| --- | --- | --- | --- | --- | --- | --- |
|  | (n=4264) | (n=1587) | (n=1070) | (n=802) | (n=805) |  |
| Age (yr),  median (IQR) | 60.4  (44.5, 72.3) | 60.9  (46.1, 73.0) | 60.3  (44.4, 72.3) | 60.2  (44.0, 71.8) | 59.7  (43.3, 71.6) | 0.116 |
| Race, n (%) |  |  |  |  |  | 0.940 |
| White | 2551 (59.8) | 964 (60.7) | 635 (59.3) | 477 (59.5) | 475 (59) |  |
| Black | 308 ( 7.2) | 105 (6.6) | 81 (7.6) | 61 (7.6) | 61 (7.6) |  |
| Other | 1405 (33.0) | 518 (32.6) | 354 (33.1) | 264 (32.9) | 269 (33.4) |  |
| Gender, n (%) |  |  |  |  |  | 0.487 |
| Female | 1781 (41.8) | 639 (40.3) | 460 (43) | 341 (42.5) | 341 (42.4) |  |
| Male | 2483 (58.2) | 948 (59.7) | 610 (57) | 461 (57.5) | 464 (57.6) |  |
| Comorbidities, n (%) |  |  |  |  |  |  |
| Comorbidities | 2578 (60.5) | 989 (62.3) | 639 (59.7) | 475 (59.2) | 475 (59) | 0.288 |
| COPD | 230 ( 5.4) | 94 (5.9) | 54 (5.0) | 42 (5.2) | 40 (5.0) | 0.694 |
| Hypertension | 1905 (44.7) | 727 (45.8) | 475 (44.4) | 351 (43.8) | 352 (43.7) | 0.700 |
| Diabetes | 846 (19.8) | 327 (20.6) | 208 (19.4) | 156 (19.5) | 155 (19.3) | 0.815 |
| Heart failure | 819 (19.2) | 302 (19.0) | 203 (19.0) | 156 (19.5) | 158 (19.6) | 0.979 |
| Laboratory parameters |  |  |  |  |  |  |
| Far,  median (IQR) | 81.4  (56.4, 126.6) | 81.5  (55.6, 128.8) | 81.1  (56.5, 125.9) | 81.1  (56.9, 126.4) | 82.9  (57.0, 127.1) | 0.933 |
| Fibrinogen (mg/dL) ,  median (IQR) | 268.5  (189.0, 398.0) | 266.0  (187.0, 394.0) | 268.0  (192.0, 398.0) | 269.5  (188.2, 398.0) | 272.0  (190.0, 399.0) | 0.842 |
| Albumin (g/dL),  mean (SD) | 3.2 ± 0.7 | 3.2 ± 0.7 | 3.3 ± 0.7 | 3.2 ± 0.7 | 3.2 ± 0.7 | 0.787 |
| Creatinine (mg/dL) ,  median (IQR) | 0.9  (0.7, 1.2) | 1.0  (0.7, 1.2) | 0.9  (0.7, 1.1) | 0.9  (0.7, 1.1) | 0.9  (0.7, 1.1) | <0.001 |
| Platelets (K/uL) ,  median (IQR) | 203.0  (141.0, 273.0) | 197.0  (134.0, 269.0) | 203.0  (145.0, 274.0) | 208.0  (145.0, 276.0) | 209.0  (146.0, 278.0) | 0.014 |
| WBC (K/uL),  median (IQR) | 12.0  (8.2, 16.9) | 11.7  (8.1, 16.9) | 11.9  (8.2, 16.7) | 12.2  (8.3, 16.9) | 12.2  (8.3, 17.0) | 0.721 |
| PT,  median (IQR) | 13.7  (12.1, 16.8) | 13.8  (12.2, 17.3) | 13.6  (12.1, 16.5) | 13.6  (12.0, 16.4) | 13.7  (12.0, 16.4) | 0.075 |
| APTT,  median (IQR) | 30.2  (26.2, 38.0) | 30.7  (26.5, 39.4) | 29.9  (26.0, 37.2) | 29.9  (26.0, 37.1) | 29.8  (26.0, 37.1) | 0.051 |
| Glucose (mg/dL),  median (IQR) | 135.0  (109.0, 176.0) | 134.0  (108.0, 177.0) | 134.0  (109.0, 173.8) | 136.0  (111.2, 177.0) | 136.0  (110.0, 176.0) | 0.687 |
| MBP (mmHg), mean (SD) | 85.3 ± 19.2 | 84.7 ± 19.2 | 85.4 ± 19.4 | 85.8 ± 19.0 | 85.8 ± 19.3 | 0.387 |
| Charlson score,  median (IQR) | 4.0  (2.0, 6.0) | 4.0  (2.0, 6.0) | 4.0  (2.0, 5.8) | 4.0  (2.0, 6.0) | 4.0  (2.0, 5.0) | 0.348 |
| SOFA score,  median (IQR) | 3.0  (2.0, 4.0) | 3.0  (2.0, 5.0) | 3.0  (2.0, 4.0) | 3.0  (2.0, 4.0) | 3.0  (2.0, 4.0) | <0.001 |
| SAPSII score,  median (IQR) | 36.0  (28.0, 45.0) | 37.0  (29.0, 47.0) | 35.0  (28.0, 43.0) | 36.0  (28.0, 44.0) | 35.0  (27.0, 43.0) | <0.001 |
| Vasopressor use, n (%) | 2558 (60.0) | 959 (60.4) | 632 (59.1) | 485 (60.5) | 482 (59.9) | 0.899 |
| MV use, n (%) | 3480 (81.6) | 1243 (78.3) | 861 (80.5) | 688 (85.8) | 688 (85.5) | <0.001 |
| Clinical outcomes |  |  |  |  |  |  |
| RRT, n (%) | 177 ( 4.2) | 125 (7.9) | 26 (2.4) | 15 (1.9) | 11 (1.4) | <0.001 |
| Hospital LOS (days),  median (IQR) | 10.9  (6.7, 19.4) | 9.1  (5.5, 17.1) | 10.8  (6.7, 18.7) | 12.7  (8.0, 21.2) | 12.8  (8.0, 21.2) | <0.001 |
| ICU LOS (days),  median (IQR) | 5.5  (3.4, 10.4) | 4.3  (2.3, 8.8) | 5.2  (3.2, 10.1) | 6.6  (4.3, 12.0) | 6.6  (4.4, 12.0) | <0.001 |
| ICU Mortality, n (%) | 556 (13.0) | 253 (15.9) | 122 (11.4) | 94 (11.7) | 87 (10.8) | <0.001 |
| Hospital Mortality, n (%) | 614 (14.4) | 276 (17.4) | 135 (12.6) | 104 (13.0) | 99 (12.3) | <0.001 |
| 30-day Mortality, n (%) | 781 (18.3) | 339 (21.4) | 178 (16.6) | 134 (16.7) | 130 (16.1) | 0.001 |
| DIC, n (%) | 100 (2.3) | 52 (3.3) | 18 (1.7) | 16 (2.0) | 14 (1.7) | 0.020 |
| AKI, n (%) | 568 (13.3) | 384 (24.2) | 103 (9.6) | 39 (4.9) | 42 (5.2) | <0.001 |

*1: No SA-AKI after ICU day 1; SA-AKI onset within ICU day 1. *2: No SA-AKI after ICU day 2; SA-AKI onset within ICU day 2. *3: No SA-AKI after ICU day 3; SA-AKI onset within ICU day 3. *4: No SA-AKI after ICU day 3; SA-AKI onset after ICU day 3. Comorbidities: any of COPD, hypertension, diabetes and heart failure. COPD: chronic obstructive pulmonary disease, FAR: the ratio of fibrinogen to albumin, WBC: white blood cell count, PT: prothombin time, APTT: activated partial thromboplastin time, MBP: mean blood pressure, SOFA: sepsis-related organ failure assessment, SAPSII: simplified acute physiology score II, MV: mechanical ventilation, RRT: renal replacement therapy, DIC: disseminated intravascular coagulation, AKI: acute kidney injury.
